# Mask wearing affects emotion perception

**DOI:** 10.1177/20416695221107391

**Published:** 2022-06-26

**Authors:** Carmel A. Levitan, Isabelle Rusk, Danielle Jonas-Delson, Hanyun Lou, Lennon Kuzniar, Gray Davidson, Aleksandra Sherman

**Affiliations:** Occidental College, 1600 Campus Road, Los Angeles, California, 90041, United States

**Keywords:** emotion perception, mask wearing

## Abstract

To reduce the spread of COVID-19, mask wearing has become ubiquitous in much of the world. We studied the extent to which masks impair emotion recognition and dampen the perceived intensity of facial expressions by naturalistically inducing positive, neutral, and negative emotions in individuals while they were masked and unmasked. Two groups of online participants rated the emotional intensity of each presented image. One group rated full faces (N=104); the other (N=102) rated cropped images where only the upper face was visible. We found that masks impaired the recognition of and rated intensity of positive emotions. This happened even when the faces were cropped and the lower part of the face was not visible. Masks may thus reduce positive emotion and/or expressivity of positive emotion. However, perception of negativity was unaffected by masking, perhaps because unlike positive emotions like happiness which are signaled more in the mouth, negative emotions like anger rely more on the upper face.

## Introduction

Understanding others’ facial expressions is an important part of our daily interpersonal communication. Effective social communication relies on recognizing others’ emotions during a conversation or interaction (e.g., recognizing that someone is happy), and is a means of revealing one's own internal emotional states to the listener (e.g., by smiling at another individual). Since the onset of the global COVID-19 pandemic in January 2020, mask wearing has become ubiquitous internationally. Mask wearing has not only been crucial for curbing the spread of the virus, but it has also increased our capacity to safely interact with one another. However, because masks cover 60%–70% of the face, obscuring the nose, mouth, cheeks, and portions of the face under the eyes, emotion expression and recognition during social interaction may be compromised.

Indeed, recent studies have demonstrated that face masks negatively affect hearing, understanding, speaker engagement, and connection (Calbi et al., 2021; Kastendieck et al., 2021; Saunders et al., 2021). Interpersonal connection is particularly challenging, partly because face masks dampen one's ability to express the appropriate emotion and partly because they restrict another individual's ability to detect the expressed emotion. When someone's face is occluded by a mask, people are significantly less accurate at detecting the depicted emotion, are more likely to confuse the depicted emotion with other emotions, and tend to perceive the depicted emotion as less intense than its unmasked counterpart (e.g., happy faces look less happy) (Bani et al., 2021; Carbon, 2020; Freud et al., 2020; Grundmann et al., 2021; Kret et al., 2021; Langbehn et al., 2020; Noyes et al., 2021; Tsantani et al., 2022). As a result, processes such as facial mimicry, which are crucial for social interaction, are impaired when one is interacting with a masked individual (Kastendieck et al. 2021).

Masking affects the perception of some expressions more than others, as certain parts of the face are more important for different emotions. For example, when expressing happiness, the mouth and lips are crucial. In contrast, the eye region exhibits the most prominent changes when expressing fear or anger, with the eyelid raising or tightening to express fear or anger, respectively (Eisenbarth & Alpers, 2011; Gagnon et al., 2014; Wegrzyn et al., 2017). Thus, emotions that rely heavily on the lower part of the face, such as happiness, disgust, and sadness, are more difficult to discriminate when masks are worn than emotions that rely more on the upper portion of the face, such as anger and surprise (Bani et al., 2021; Langbehn et al., 2020; Noyes et al., 2021; Tsantani et al., 2022). The idea that our ability to discriminate between emotions is enhanced when we have visual access to the entire face, is consistent with theories about holistic processing of faces. Specifically, evidence shows that participants are slower and less accurate at detecting emotional states when presented with only the top or bottom half of the face (e.g., Calder et al., 2000). Meanwhile, neutral expressions tend to be recognized accurately in both masked and unmasked conditions (Noyes et al., 2021).

Although masking may impair the expression and perception of emotions, it is possible that people's exposure to mask wearing over the past 2 years has resulted in changes in communication, emotion expressivity while masked, and perception of emotions. In a recent study conducted in the UK, 60% of individuals reported communicating differently when wearing face coverings (Saunders et al., 2021). In order to be better understood, people may overcompensate by increasing the volume and expressivity of their speech, using body language such as gestures, and attempting to communicate through their eyes (Saunders et al. 2021). Increasing emotional signals in the eyes would decrease the negative effects of masking on emotion perception. It is also possible that increased mask exposure has led to a more nuanced perception of emotions, meaning that people with more mask exposure may be able to better discriminate emotions from the eyes, even when the signal remains relatively unchanged. Barrick et al., 2021 support this, finding that when completing an emotion recognition and similarity task, participants with increased mask exposure used cues from the eyes to a greater extent than those with less mask exposure. These findings suggest that mask wearing is shifting the way that emotions are recognized from more holistic processing of the entire face to extracting signals from a more localized area such as the eyes and forehead.

The present study aims to replicate and extend the recent work on emotion expression and perception while masking. We were interested in whether mask wearing causes people to exaggerate their emotions and increase the emotion-related information in their eyes. We were particularly interested in the differential effects that mask wearing may have on the expression of happiness, which relies primarily on the mouth, and on the expression of anger, which relies primarily on the eyes. Whereas many previous studies use emotional face stimuli that are produced by actors, we induced negative and positive emotions in a more naturalistic setting via Zoom interviews with non-actors. Moreover, several previous studies investigating the effects of masking on emotion perception digitally overlay a mask onto a face stimulus. However, because we hypothesized that an individual might exaggerate their emotions when masked, we induced emotions both when participants were masked and unmasked. Second, we were interested in the extent to which mask exposure and social interaction might affect emotion expression perception. We predicted that people with greater mask exposure would be more accurate at recognizing both positive and negative emotions from the eyes alone.

## Method

### Overview

We conducted a mixed 3  × 2  × 2 experiment with within-participants variables of emotion (positive, negative, and neutral) and masking (masked and unmasked face stimuli), and a between-participants variable of cropping (whether the full face was visible or just a cropped image of the upper face). Participants rated the valence of the faces, and we conducted two sets of analyses of this data. In the first analysis, we used the raw valence as a dependent variable. In the second set of analyses, we used the valence ratings to calculate accuracy for the ratings of the positive and negative faces. All data and stimuli are available on OSF (https://osf.io/bjuqm/).

### Participants

We recruited 206 participants via Prolific Academic (98 women, 79 men, and 29 no gender reported due to an error in presenting the questions; the most common countries represented in our sample were Mexico, South Africa, United States, United Kingdom, and Portugal). They were compensated $5. The Human Subjects Research Review Committee at Occidental College (HSRRC-IRB) approved the study.

### Stimuli

**Figure 1.**
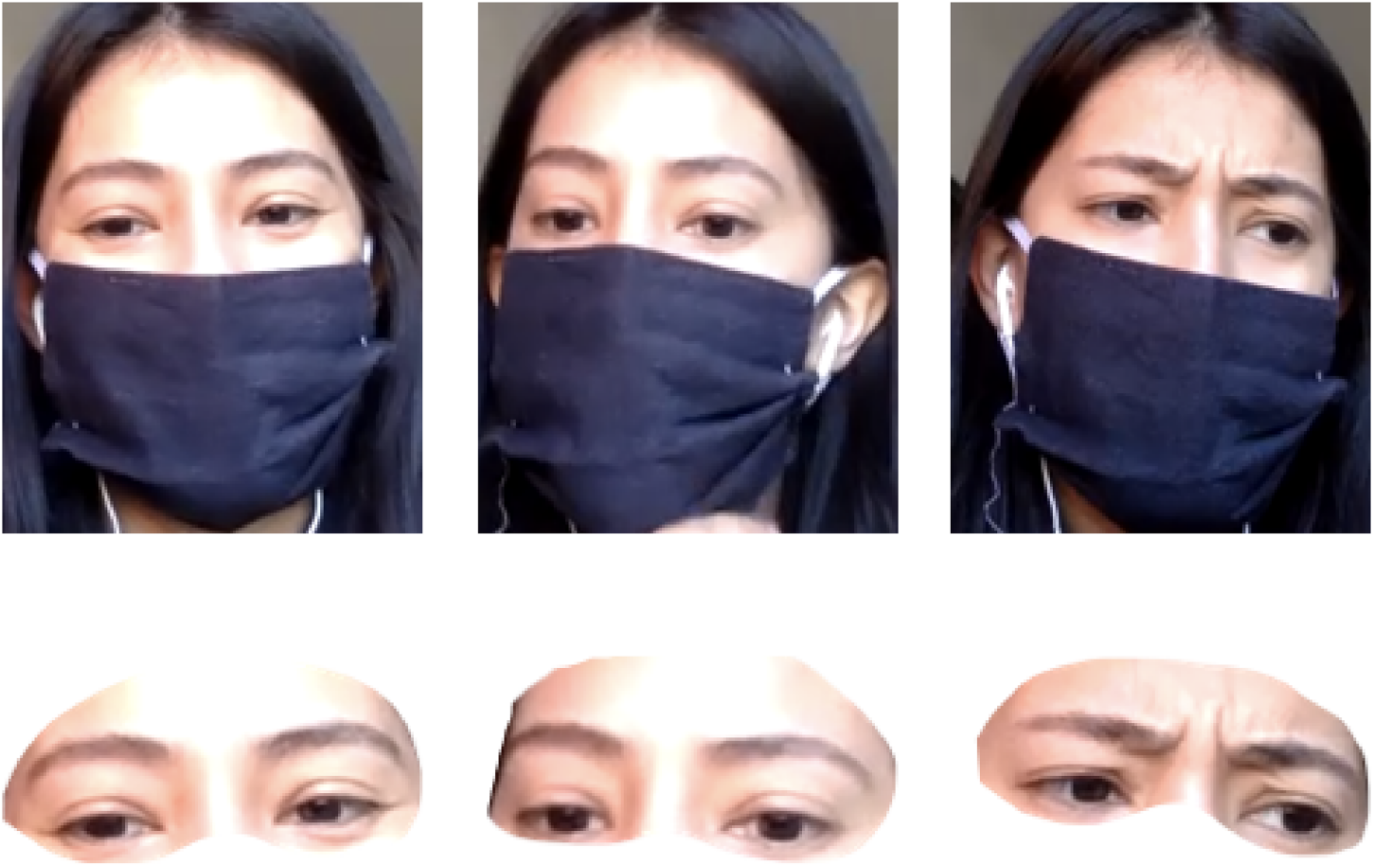
Sample masked and cropped images displaying positive, neutral, and negative conditions, respectively.

We created the stimuli by inducing negative and positive emotions in a group (N=93) of undergraduate students via Zoom while they were both masked and unmasked. Each participant was interviewed by an experimenter and asked to respond to prompts designed to elicit positive, neutral, or negative emotions (e.g., positive: “Talk about a time when you did something kind for another person or when someone did something nice for you. How did they react? How did you feel?”, neutral: “What are you currently reading for class?”, and negative: “Describe a negative situation, caused by someone else, in which you experienced an extremely intense emotional response. For example, a time you have been cheated, lied to, labeled, or blamed unfairly. How did or does that make you feel?”). They answered one set of questions while masked, and a different set of questions while unmasked; order was randomized across participants for both the variables of masking and emotional valence. Interviews were conducted in a consistent way by three of the undergraduate coauthors. The interviews were then assessed frame-by-frame by multiple members of the research team to identify moments of most intense expression for each participant in each of the six conditions. For all expression types (positive/negative/neutral), at least two frames were selected per individual. The selected frames were edited to focus on the face. These images were used in the uncropped condition. For the cropped condition, the images were further edited so that the regions of the face that normally would be covered by a mask were cropped out. To ensure that participants would not be able to determine whether the depicted individual was masked or unmasked, all images were cropped in the same way. To select the final stimulus set, five members of the research team rated images by categorizing them as positive, negative, or neutral, as well as to rate how intense the emotion was. Only stimuli that were correctly classified by at least three team members for all three expression types were included in the final stimulus set. The final stimulus set included 19 unique individuals (gender: 3 males, 13 females, 3 nonbinary; race/ethnicity: 6 Asian, 6 Latinx, 2 Black, 2 White) expressing positive, neutral, and negative emotions while masked and unmasked, with a total of 228 images (114 cropped and 114 uncropped). Figure 1 shows sample stimuli. The final stimulus set is available on OSF (https://osf.io/74jbu/).

### Procedure

Participants (N=102 for cropped condition; N=104 for uncropped condition) gave informed consent, and then were instructed to rate the valence of each emotional face using a scale that ranged from −10 to 10, with −10 labeled as negative, 0 labeled as neutral, and + 10 labeled as positive. They then answered a survey about their own mask exposure and wearing, and finally answered demographic questions. For a small number of participants, the mask exposure questions were not initially displayed, but the majority of those participants answered the additional questions the same day for an extra $1.25 in compensation. Most participants completed the study in less than 20 min.

## Results

We assessed how masking affected expression perception of positive, negative, and neutral faces. Our primary dependent measures were valence ratings, measured on a scale from −10 (most negative) to + 10 (most positive), and accuracy (measured as the percentage of correct responses; this metric was not applied to neutral faces). A response was marked as correct when participants’ valence ratings aligned with the intended expression valence. For example, if participants were shown an image of a face expressing a positive emotion, any rating above 0 would be marked as correct, whereas a rating below 0 would be marked as incorrect. Average valence ratings and accuracy scores across all stimuli in each category were computed for each participant. Statistics were conducted using JASP (https://jasp-stats.org/).

**Figure 2.**
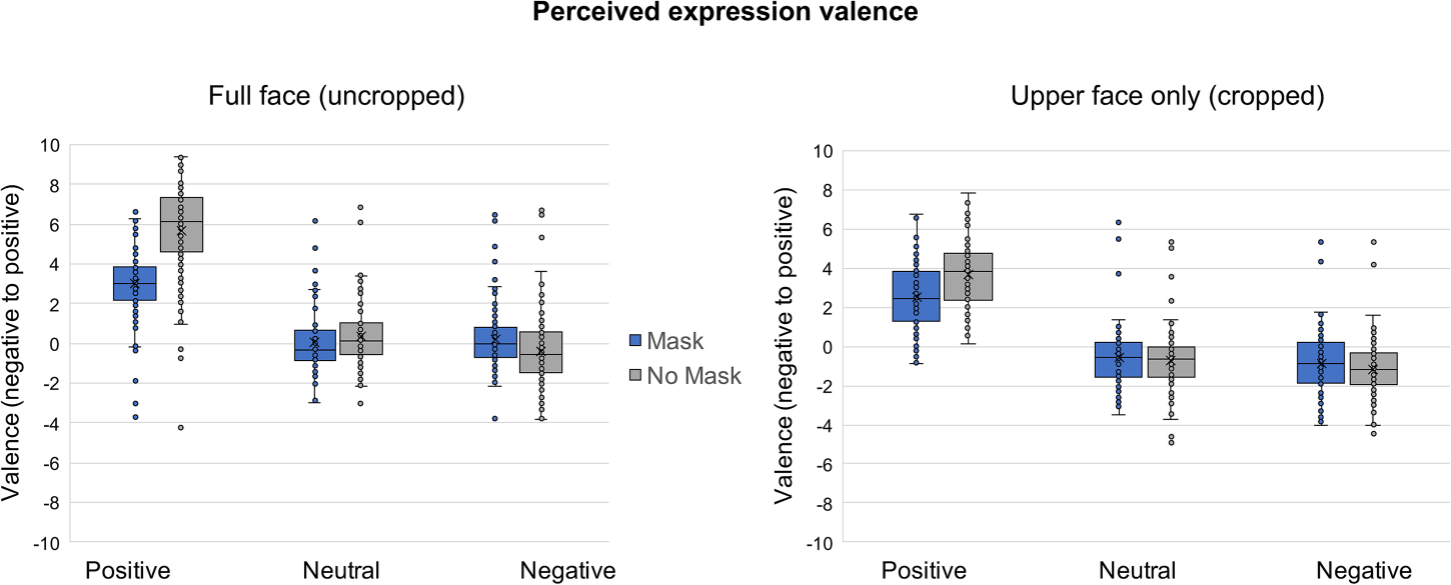
Perceived valence for uncropped (left panel) and cropped (right panel) positive, neutral, and negative faces. Blue bars (left bars) are masked faces and gray bars (right bars) are unmasked faces.

For valence ratings, we ran a mixed 3  × 2 × 2 ANOVA with emotion (positive, neutral, and negative) and masking (mask, no mask) as within-subjects variables and cropping (full face, upper face only) as a between-subjects variable (Figure 2). Where sphericity assumptions were not met, we report Greenhouse–Geisser corrections. First, there was a main effect of emotion (*F*(1.191, 203) = 694.444, *p* < .001, $\eta_{p}^{2}$ = 0.773) such that positive faces were rated as significantly more positive than both neutral and negative faces (*t*(204) > 30.95, *p*_holm_< 0.001, Cohen's *d* >2.16). Although negative faces were rated as significantly more negative than neutral faces (*t*(204)=2.53, *p*_holm_ = 0.012, Cohen's *d* = 0.18), the effect was quite small. This could be because negative emotions like anger were harder to induce or that naturalistic negative emotions like anger are a more subtle expression than naturalistic happiness. There was also a significant three-way interaction (*F*(1.809, 368.958) = 41.78, *p* < .001, $\eta_{p}^{2}$ = 0.170). Of particular interest is that positive faces were the most affected by masking. As predicted, participants rated the valence of positive masked faces similarly whether or not they were shown the full face which included the mask, or the upper face only where they were unaware that a mask was being worn (t(204) = 1.812, *p*_holm_=0.602, M_diff_ = −0.434, 95% CI [−1.245, 0.377]). However, unmasked positive faces were rated as significantly less positive when only the upper face was shown relative to when the full face was shown (t(204) = 8.320, *p*_holm_ < 0.001, M_diff_ = −1.993, 95% CI [−2.804, −1.183]); this is consistent with the notion that the mouth conveys a portion of the positive signal. Crucially, masking dampened expression perception of positive faces both when full faces were shown (t(204) = 20.967, *p*_holm_ < .001, M_diff_ = −2.686, 95% CI [−3.120, −2.252] and when only the upper face was shown (t(204) = 8.709, *p*_holm_ < .001, M_diff_ = −1.216, 95% CI [−1.565, −.688]). Since participants in the upper face only condition were unaware of whether the person was masked or not, the fact that there is a significant difference between the two masking conditions suggests that the amount of positive signal within the eyes varies depending on if the depicted person was masked. In contrast to our hypothesis that masking may increase expressivity, these data suggest that masking actually makes people less expressive (or potentially less happy). Neutral and negative faces were not impacted by masking or cropping in the same way. This is consistent with past results that suggest much of the perception of happy faces is contained within the lower half of the face in the smile, whereas anger is more in the eyes, forehead, and eyebrows.

**Figure 3.**
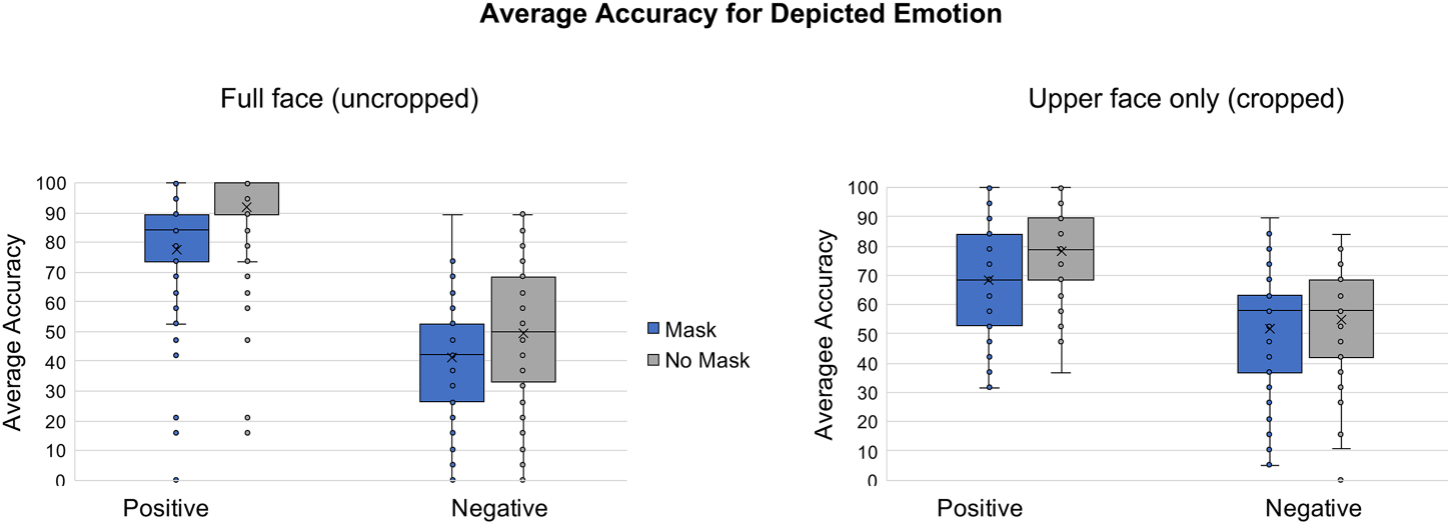
Mean accuracy for uncropped (left panel) and cropped (right panel) positive and negative faces. Blue bars (left bars) are masked faces and gray bars (right bars) are unmasked faces.

For average accuracy scores, we ran a mixed 2  × 2 × 2 ANOVA with emotion (positive, negative) and masking (mask, no mask) as within-subjects variables and cropping (full face, upper face only) as a between-subjects variable (Figure 3). Again, there was a significant main effect of emotion such that positive faces were correctly identified more often than negative faces (*F*(1, 204) = 333.28, *p* < .001, $\eta_{p}^{2}$ = 0.620). This is consistent with the valence ratings, showing that there was more confusion in rating and identifying negative emotions than positive emotions. There was also the main effect of masking such that masked faces were more difficult to identify than unmasked faces, regardless of emotion (*F*(1, 204) = 164.697, *p* < .001, $\eta_{p}^{2}$ = 0.447). Similarly to the valence ratings, we observed a significant interaction between emotions and masking (*F*(1, 204) = 13.556, *p* < .001, $\eta_{p}^{2}$ = 0.462) indicating that masking affected accuracy more for positive faces than for negative faces. Moreover, an interaction between masking and cropping (*F*(1, 204) = 14.127, *p* < .001, $\eta_{p}^{2}$ = 0.065) demonstrates that masking led to accuracy differences moreso when full faces were shown. There was no significant three-way interaction (*F*(1, 204) = 0.022, *p*=.882).

We had originally hypothesized that participants who are exposed to more mask wearing might be better at extracting emotional information from cropped and masked faces than participants with less exposure to masked faces. Interestingly, the participants in the full face (cropped) compared to the upper-face only (uncropped) experiments had very different reports of their own mask use and that of others. Specifically, those in the full face experiment reported more mask wearing than those in the upper face only experiment, though other demographics of the samples were similar (age, country, gender, and ethnicity). This may suggest that exposure within the experiment to people wearing masks influences people's retrospective memories, such that seeing others in masks leads you to overestimate your own mask wearing habits. We thus did not further investigate the relationship between reports of mask-wearing and emotional perception.

## Discussion

The present study investigated the extent to which masks impair emotion recognition and the intensity of perceived expressions. Because of the increased prevalence of masks, we hypothesized that people would compensate for the emotion-dampening nature of masks by exaggerating their expressions in their upper face. To test this, we asked participants to rate the emotional intensity of naturalistic images depicting masked and unmasked individuals expressing positive, negative, and neutral emotions. Whereas previous research exclusively used images or videos of masks superimposed on individuals expressing specific emotions, we created our stimuli by inducing emotions in individuals over Zoom while they were masked and unmasked. This approach has the advantage of better characterizing people's natural emotional expressions while masked, especially as face coverings may on their own inhibit expression production. Moreover, we validated that participants’ emotion ratings were not biased by the knowledge of whether the individual was masked by comparing ratings of full-face images to cropped images depicting only the upper face.

We found that masks impaired the recognition of and intensity of positive, but not negative emotions. This effect was evident both when the full face was shown and when participants only saw the upper face and could not determine whether the individual was masked or unmasked. Contrary to our hypothesis, people wearing masks did not compensate by exaggerating their emotional expressions; instead, masked positive faces were actually rated as less positive than unmasked positive faces even when the lower part of the face was not visible. This could be due to masks dampening positive emotion (e.g., people feel less happy when wearing a mask), and/or to masks reducing expressivity of positive emotion (e.g., facial expressions might be diminished while wearing a mask). Thus, mask wearing might lead to both reduced expression of positive emotion as well as underestimation of positive emotion. The fact that masks did not affect perception of negative faces is also consistent with prior research. In particular, prior work suggests masks affect emotions like happiness because it relies on the mouth more than anger and disgust which rely on the eyes and eyebrows. Another possibility is that we didn’t see an effect of masking because of the specific stimuli we employed. Notably, the negative emotion ratings were less intense than the positive ratings, and participants were significantly less accurate in categorizing negative emotions relative to positive ones, irrespective of masking. This could mean that our method for inducing negative emotions was less powerful than our method for inducing positive emotions. Alternatively, it could be that naturalistic, everyday negative emotions like anger are more subtle than naturalistic happiness. It is possible that with a more robust induction of negative emotions, we would see a more robust effect of masking.

There are also additional interesting avenues worth expanding on that were outside of the scope of the present study. First, it is possible that people do exaggerate their emotional expressions, but this effect is modulated by social factors such as increased exposure to masks, increased likelihood of wearing masks while engaged in social interactions, and increased empathy and compassion for others. Recent research suggests that increased exposure to masks improves people's ability to perceive emotional expressions in others (Barrick et al., 2021) and this might similarly extend to emotion production. Second, dynamic displays rather than static images may show the effects of masking on both production and perception more robustly. Computational techniques using dynamic displays might be especially useful in discriminating small signal differences between masked and unmasked expressions (e.g., Murugappan & Mutawa, 2021).
